# Cognitive interviewing to improve women's empowerment questions in surveys: Application to the health and nutrition and intrahousehold relationships modules for the project‐level Women's Empowerment in Agriculture Index

**DOI:** 10.1111/mcn.12871

**Published:** 2019-08-14

**Authors:** Anika Hannan, Jessica Heckert, Laurie James‐Hawkins, Kathryn M. Yount

**Affiliations:** ^1^ Hubert Department of Global Health, Rollins School of Public Health Emory University Atlanta Georgia USA; ^2^ ICF, International Health and Division Rockville Maryland USA; ^3^ International Food Policy Research Institute Washington District of Columbia USA; ^4^ Department of Sociology University of Essex Colchester UK; ^5^ Department of Sociology Emory University Atlanta Georgia USA

**Keywords:** Bangladesh, cognitive interviewing, gender, survey methods, women's agency, women's empowerment

## Abstract

In 2015, the United Nations adopted the Sustainable Development Goals, which include fostering gender equality and women's empowerment and ending hunger and malnutrition. To monitor progress and evaluate programmes that aim to achieve these goals, survey instruments are needed that can accurately assess related indicators. The project‐level Women's Empowerment in Agriculture Index (pro‐WEAI) is being developed to address the need for an instrument that is sensitive to changes in empowerment over the duration of an intervention. The pro‐WEAI includes new modules with previously untested survey questions, including a health and nutrition module (focused on women's agency in this area) and an intrahousehold relationships module. This study uses cognitive interviewing to identify how new survey questions might be misinterpreted and to understand what experiences women are referencing when they respond to these questions. This was undertaken with the goal of informing revision to the modules. The study was conducted in Bangladesh with women from nuclear, extended, and migrant‐sending households and from two regions of the country to identify difficulties with interpretation and response formulation across these groups. Findings revealed that questions were generally understood, but participants occasionally responded to the wrong part of the question, did not understand key phrases, or were uncomfortable with questions. The findings also suggested ways to revise the modules and strengthen the pro‐WEAI. The revised pro‐WEAI health and nutrition and intrahousehold relationships modules will advance the ability to measure changes in these domains and their relationship with the health and nutritional status of women and their children.

**Key messages**

- To monitor the progress of the Sustainable Development Goals and advance research on the complex relationship between women's empowerment and health and nutrition, rigorous approaches should be applied to the development of instruments to measure women's empowerment.
- Cognitive interviewing revealed that questions in the two modules being tested were generally understood but also highlighted that the structure of some questions and unfamiliar phrases made them difficult to understand.
- Responses from women with migrant husbands suggest that sole decision‐making may indicate a lack of support or that they consult nonhousehold members on key decisions.

## 1 INTRODUCTION

In 2015, the United Nations adopted the Sustainable Development Goals. These 17 goals include ending hunger and malnutrition (Goal 2) and improving gender equality and women's empowerment (Goal 5; United Nations General Assembly, 2015). As part of monitoring progress towards achieving these goals, valid survey instruments to measure women's empowerment are needed (Hindin, 2000; Shroff, Griffiths, Adair, Suchindran, & Bentley, 2009). Valid survey instruments are also critical for understanding the links between interrelated targets, such as women's empowerment, gender equality, and reduced hunger and malnutrition.

The project‐level Women's Empowerment in Agriculture Index (pro‐WEAI) is being developed to address the need for an instrument that is sensitive to changes in women's empowerment over the course of an agricultural development project. The pro‐WEAI adapts and extends the Women's Empowerment in Agriculture Index for this purpose (Alkire et al., 2013; Malapit, Sproule, & Kovarik, 2017). The pro‐WEAI focuses on the agency necessary for women to act on their aspirations related to agriculture (Malapit et al., 2016; Malapit et al., 2017; Malapit et al., 2019). The pro‐WEAI introduces new modules and allows for optional modules to meet programme needs. These new modules include survey questions that have not yet been widely used to measure empowerment: the intrahousehold relationships module (part of core pro‐WEAI) and the optional health and nutrition module (Heckert et al., 2018; Malapit et al., 2017; Malapit et al., 2019). The development of a health and nutrition module is motivated by an increased focus on nutrition‐sensitive agriculture, which aims to address the underlying determinants of malnutrition, often through multisectoral approaches (Ruel, Alderman, & Maternal and Child Nutrition Study Group, 2013), as well as evidence of the agency‐related pathways by which women's income generation and other enabling resources are related to improvements in women's dietary diversity and nutrition (Sinharoy et al., 2018; Sinharoy et al., 2019). Some evaluations of nutrition‐sensitive agriculture programmes have included indicators of women's empowerment related to production and to health and nutrition; however, survey instruments to measure women's empowerment in health and nutrition are not yet widely accepted or rigorously evaluated (Malapit et al., 2014; Olney et al., 2016; Ruel, Alderman,, & Maternal and Child Nutrition Study Group, 2013). Inclusion of the intrahousehold relationships module is motivated by calls from implementing partners who wish to measure the impact of these projects on intrahousehold dynamics (Malapit et al., 2019) and by recent studies that link men's engagement to improved maternal and child health outcomes (Doyle et al., 2018; Doyle, Kato‐Wallace, Kazimbaya, & Barker, 2014). To date, however, modules that measure women's nutrition‐specific agency and the quality of intrahousehold relationships have been lacking.

These modules have the potential to elucidate the relationship among agricultural development programmes, women's empowerment, and health and nutritional outcomes. Before adopting these new modules broadly, it is necessary to test them with the participant population (Crandall, Rahim, & Yount, 2015; Galié et al., 2017; Shaikh et al., 2016; Yount, VanderEnde, Dodell, & Cheong, 2016). Cognitive interviewing is a useful approach to understand whether questions are understood as intended, the motivations for responses, and whether the given responses reflect participants' experiences.

### 1.1 Conceptualizing women's empowerment

Kabeer's (1999) seminal framework presents women's empowerment as a dynamic process that entails “expansion in people's ability to make strategic life choices in a context where this ability was previously denied to them” (p. 437). The framework focuses on three interrelated dimensions: resources, agency, and achievements. Resources are enabling factors, including, but not limited to, material, human, and social resources (Kabeer, 1999; Malhotra & Schuler, 2005; Miedema, Haardörfer, Girard, & Yount, 2018; Yount et al., 2016). The new claims that women make on these enabling resources are necessary preconditions for, but do not guarantee, agency if a woman does not, as a result, develop critical consciousness to leverage these resources to fulfil her aspirations (Kabeer, 1999; Malhotra & Schuler, 2005). Agency is the ability to define one's goals and act upon them, such as through choice or negotiation (Kabeer, 1999; Malhotra & Schuler, 2005). Lastly, achievements are the outcomes related to women's welfare, which result from their exercise of agency and the fulfilment of their personal aspirations (Kabeer, 1999). Achievements cut across political, economic, social, and health‐related domains (Gram et al., 2017; Heckert & Fabic, 2013; Malhotra & Schuler, 2005). All three dimensions—resources, agency, and achievements—are constitutive and reflective of the process of empowerment. Our interest here is to expand this concept to consider a measure of women's food and nutrition‐related agency as a mediator in the relationship of women's enabling resources and their dietary diversity and nutrition. Such a measure to date has been lacking in the literature on women's empowerment and nutrition (Morgan, 2016).

### 1.2 Cognitive interviewing of pro‐WEAI modules

The dimensions of empowerment related to agency are abstract and not easily observed. To capture abstract concepts, such as agency in health and nutrition, requires the use of multiple questions, and it is important to ensure that these questions are interpreted as intended and that the response options resonate with the answers that participants give naturally. Cognitive interviewing is a qualitative method to assess participants' understanding of survey questions (Malapit et al., 2014; Willis, 2004; Willis & Miller, 2011). Discrepancies between how questions are asked and interpreted can occur at any stage of the cognitive process of interpretation, recall, motivation, and response, resulting in response error (Willis & Miller, 2011). Cognitive interviewing can identify potential sources of error and provide insight into participants' interpretation of survey questions. Cognitive interviewing also can reveal how heterogeneous subgroups of participants may interpret survey questions differently, and this information can be used to help maintain content validity across diverse populations (Gram et al., 2017; Willis & Miller, 2011). Overall, findings from cognitive interviewing can inform revisions to questions and questionnaire modules that improve the overall quality of the instrument.

To identify potential errors at each stage of the cognitive response process, cognitive interviewing assesses four cognitive processes: comprehension, retrieval, judgment, and response (Malapit et al., 2016; Yount, Halim, Schuler, & Head, 2013; Willis, 2004). Comprehension is the participant's understanding of the question's content and key terms and includes what the participant recalled when answering the question. Retrieval evaluates if a participant can accurately recall the content needed from a specific time period to answer the question. Judgment determines if participants might feel uncomfortable with content. Response determines whether the participant can easily respond to the question in the format suggested.

### 1.3 Purpose of study

This study aims to inform revisions to the pro‐WEAI health and nutrition and intrahousehold relationships modules to produce an improved data collection instrument that can advance our understanding of the important relationship between women's empowerment and health and nutrition. The specific aims of the study were twofold: (a) identify areas of potential error based on four aspects of the cognitive processes undertaken while responding to survey questions (comprehension, retrieval, judgment, and response) and (b) understand the context and specific experiences that women considered when answering each question. We examine these issues among mothers with children under age 2 in two regions of Bangladesh and in three household types: (a) nuclear households (husband and wife with no coresiding parents), (b) intergenerationally extended households (husband and wife living with family members that include the husband's mother, referred to as extended from here forward), and (c) migrant‐sending households (a wife with a husband who was absent due to labour migration) to understand to what extent women from different subgroups interpreted or responded to decision‐making questions differently.

## 2 METHODS

### 2.1 Study setting and context

The study was conducted in Bangladesh, one of the countries where the pro‐WEAI is being used to evaluate nutrition‐sensitive agriculture projects. Despite considerable change in Bangladesh, family relationships and gender roles remain patriarchal, and women's social position is defined in relation to men through marriage and family (Kandiyoti, 1988). In Bangladesh, the extended family household structure remains common, with a married woman living with her husband's immediate family (Kabeer, 2011; Samad, 2015). Women living in extended household structures may have less agency compared with those living in nuclear household structures and must seek permission from multiple household members to undertake certain activities (Debnath, 2015).

In Bangladesh, labour migration influences household structure and women's agency (Hadi, 2001; Rahman, 2009). Annually, 500,000 Bangladeshis, primarily men, travel to Middle Eastern or other Southeast Asian countries for work (Asian Development Bank, 2016). In her husband's absence, the wife may assume the role of the family head, receive the money sent home by her husband, and exercise a primary role in family decision‐making (Rahman, 2009). This new social position may result in increased access to resources, greater self‐confidence, and greater freedom of movement (Hadi, 2001). In other cases, a father or brother‐in‐law may serve as a proxy, or a phone consultation with a husband may influence how decisions are made, resulting in limited expansion of women's agency.

The study site was two rural upazilas (district subunits): Sitakunda in the Chittagong division and Aditmari in the Rangpur division. Chittagong is located by the Bay of Bengal and has the lowest rate of poverty in Bangladesh, and gender attitudes in this region are more conservative (World Bank Group, 2016). Rangpur is located in northern Bangladesh, close to India, and is one of the poorest divisions (World Bank et al., 2010; World Bank Group, 2016). The two divisions differ in labour migration patterns with 13.2% of households in Chittagong having one or more family members abroad, compared with 0.6% in Rangpur (Bangladesh Bureau of Statistics, 2015).

Women living in different household types and regions may interpret questions about household decisions and construct their responses to these questions differently. For example, a woman might not know how to describe her husband's involvement in decision‐making if he is away (Debnath, 2015). Purposefully interviewing women from different household types and regions allows for better understanding of how women in various circumstances respond to questions.

### 2.2 Participants and procedures

Data Analysis and Technical Assistance Limited (DATA), a research consulting firm in Bangladesh, partnered on the data collection. DATA translated interview guides to Bengali, recruited and interviewed participants, translated the Bengali responses into English, and compiled that data into a format that could be analysed by the authors. The first author worked closely with DATA during translation, trained the field team, and oversaw fieldwork.

The field team recruited mothers with children younger than age 2 years who lived in one of the three household types: nuclear, extended, or migrant‐sending. Interviewers used snowball‐sampling methods to identify additional households that met the inclusion criteria. Interviews took place in or immediately outside the women's home. Participants were interviewed privately, away from other household members, and field staff was trained to mitigate interruptions. The sample size commonly used for cognitive interviewing ranges from five to 15 interviews for each subgroup of interest (Beatty & Willis, 2007; ). A total of 48 interviews were conducted: 16 from each household type, divided equally between the two study sites.

### 2.3 Ethics

The institutional review board of Emory University approved this study. Participants provided written informed consent and were compensated with two melamine plates and a bowl, valued at approximately 2.50 USD.

### 2.4 Pro‐WEAI modules

The health and nutrition module was divided into four sections. The first three sections asked about decisions related to 30 different activities: (a) women's health and nutrition (e.g., how much you can rest when you are ill; six questions), (b) women's health and nutrition during pregnancy and breastfeeding (11 questions), and (c) child health and nutrition (e.g., whether your child gets vaccinations; 13 questions; full content available in Table S1). For each topic, they were asked:

1. “Who in the household generally makes decisions about [ACTIVITY]?” to which she could list up to three individuals, including herself.
2. “To what extent do you feel you can participate in decisions regarding [ACTIVITY] if you want (ed) to?” to which she could respond “not at all,” “small extent,” “medium extent,” or “high extent.”
3. “Who would you prefer make the decisions about [ACTIVITY]?” to which she could list up to three individuals, including herself.

In the fourth section, women were asked about obtaining 12 necessities (types of foods, health products, clothing, and toiletries). They were asked:

1. “Who in the household generally makes decisions about whether to purchase [PRODUCT]?” to which she could list up to three individuals, including herself, and
2. “If you need [PRODUCT], are you usually able to acquire it by some means (e.g., purchasing or cultivating it yourself or having someone do it for you)?” to which she could respond “yes,” “no,” or “not applicable.”

The intrahousehold relationships module asked about a woman's relationship with her husband and mother‐in‐law. Participants were asked the following questions, to which they could respond “never,” “rarely,” “sometimes,” or “most of the time.”

1. “Do you [NAME] respect your [RELATION]?”
2. “Does your [RELATION] respect you?”
3. “Do you trust your [RELATION] to do things that are in your best interest?”
4. “When you disagree with your [RELATION], do you feel comfortable telling him/her that you disagree?”

### 2.5 Cognitive interview guide

The cognitive interview guide was adapted from a guide developed to cognitively test the original WEAI (Johnson & Diego‐Rosell, 2015; Malapit et al., 2016). Scripted probing questions were used to minimize enumerator error (Willis, 2004). This type of probing does not require expert pro‐WEAI knowledge and can be carried out by trained interviewers (Johnson & Diego‐Rosell, 2015). Five key probing questions were developed based on the four stages of cognitive response model (comprehension, retrieval, judgment, and response). Participants were asked about

1. Comprehension:
  - Recall period: “Some people may think of specific experiences when they hear this question, or they may think about their life in general. Were you thinking of any of the following or something else?”
  - Abstract concepts and key questions: “Can you repeat this question in your own words?” or “What specifically did you think that I meant when I said [e.g., rest]?”
2. Retrieval: “Many people find it difficult to recall activities done a long time ago. How well do you remember the type of decisions that you made while [X]?”
3. Judgment: “Think of another mother with a young child in your community. Do you think that other women you know may find it difficult to answer these questions for any reason?” If yes, they were asked, “Why do you think they may find it difficult?”
4. Response: “Did you find this question easy or difficult?” If difficult, they were asked, “Why was it difficult?” and “Did the question make you feel uneasy or uncomfortable?” If yes, they were asked, “Why was it uneasy or uncomfortable?”

For questions where it was informative to know more information on the context or whether they responded according to actual experiences, we asked:

1. Context: “Please tell me what you were thinking while you answered this question.”
2. Decision‐making process: “I'd like you to tell me about a time when [e.g., you were ill]. If a decision was made to [e.g., consult a doctor/go to a clinic], how was the decision made [e.g., whether or not to consult a doctor or go to a clinic]?”

### 2.6 Interviews

The interview began by administering a household roster. Interviewers administered each of the four sections of the health and nutrition module. Immediately after each section was administered, interviewers asked the related cognitive interviewing questions. The same format was followed for the intrahousehold relationships module.

Each interview team included an interviewer and a notetaker. Interviews were not recorded. As participants responded to the cognitive interview questions, the interviewer and notetaker noted the responses to close‐ended question and either transcribed verbatim in Bengali or noted key content for open‐ended questions, depending on the length of the responses. The notetaker observed the participant during the interview and recorded non‐verbal cues, verbal indicators of confusion or hesitancy, and information on the immediate environment related to the interview. These observations were used to supplement verbal statements and to note reactions that participants may or may not have explicitly stated. The transcribed responses were translated from Bengali into English. The first author, who is fluent in Bengali and English, identified potentially erroneous translations, cross‐checking with the original Bengali, as needed.

### 2.7 Analysis

The primary author carried out the analysis on the cognitive interview questions, with support from the tertiary author. For close‐ended questions (e.g., did you find this question easy or difficult?), data were analysed by grouping similar participant answers together. For open‐ended questions (e.g., “Why did you find this question difficult?”), the primary author used a thematic analysis approach and developed a codebook by identifying emerging themes directly from the data (see Table S3). The themes served as the framework for the codebook (Braun & Clarke, 2006). The codebook was adjusted accordingly to integrate subthemes that emerged during data analysis. Responses were compared across household type (nuclear, extended, and migrant‐sending) and division (Chittagong and Rangpur).

## 3 RESULTS

Notable results presented from the four stages of the cognitive responses model—comprehension, retrieval, judgment, and response—identify potential areas of misinterpretation in the pro‐WEAI health and nutrition and intrahousehold relationships modules. Results for the questions on the context of decision‐making also are presented to provide insight into response processes.

Participants were 24 years old, on average, and 17% had completed secondary school (Table 1). The mean age of the youngest child was 10 months. Half of participants (*N* = 24) lived in the Chittagong, whereas the other half (*N* = 24) lived in the Rangpur division. Participants were equally divided among the three household types (nuclear, extended, and migrant‐sending), which was consistent with the recruitment strategy.

**Table 1:** Characteristics of respondents and their youngest child

		Household type			Division	
	Total	Nuclear	Extended	Migrant‐sending	Chittagong	Rangpur
	*n* = 48	*n* = 16	*n* = 16	*n* = 16	*n* = 24	*n* = 24
Age (years), mean (*SD*)	24 (0.65)	26 (0.86)	23 (1.36)	23 (1.04)	24 (0.77)	24 (1.06)
Completed primary school^a^ or some secondary school, *n* (%)	23 (48%)	8 (50%)	10 (63%)	5 (31%)	13 (54%)	10 (42%)
Completed secondary school or higher, *n* (%)	16 (33%)	2 (13%)	4 (25%)	10 (63%)	8 (33%)	8 (33%)
Youngest child's age (months), mean (*SD*)	10 (0.97)	9 (1.44)	11 (1.83)	12 (1.77)	10 (1.39)	11 (1.37)

a Primary school completion of Grade 5.

### 3.1 Comprehension: Understanding of key questions and terms

A majority of the participants (72%) were able to repeat all key questions in their own words and to maintain its intended meaning. Those who interpreted the question differently than intended focused on the specific domains or activities in the question, instead of the decision‐making portion of the question (Table 2). For example, some participants interpreted the question “Who in the household generally makes decisions about how much you could rest if you were ill?” as “How long will you rest if you fall ill?”

**Table 2:** Comprehension: Summary of responses to cognitive interview questions eliciting information on the comprehension of survey questions and terminology

Decision‐making topic or question, *n* = 48	Number that did not understand intended meaning	Examples of unintended meanings in participants own words (frequency shown in parentheses)
Who in the household generally makes decisions about [decision]?	8 (17%)^a^	How long will you rest? (2); how long will you rest if you fall ill? (4)
To what extent you feel you can participate in decisions regarding [decision] if you wanted to?	10 (23%)^b^	How long will/can you rest if you fall ill? (6); whose decision do you like if you fall ill? (1); who takes the decision/how is the decision made to have another child? (2)
Who you would prefer make decisions about [decision]?	6 (13%)^a^	Who will take decision when you become sick/how much to rest? (2); how much time will you rest? (1)
Who in the household generally makes decisions about whether to purchase [product]?	6 (13%)^a^	Who buys item? (3)
If you needed [product], are you usually able to acquire it by some means (e.g., purchasing or cultivating it yourself or having someone do it for you)?	14 (30%)^a^	How will you get an item? (12)
Do you respect your husband?	3 (6%)	How much does your husband honour you? (1); how much do you honour your husband? (1)
Does your husband respect you?	2 (4%)	My husband did not like me after marriage. (1)
Do you trust your husband to do things that are in your best interest?	28 (58%)	Do you trust/believe/have faith in your husband with important/special matters? (8); do you rely on your husband for something important? (6); do you trust your husband? (5); can you share with your husband? (4); does your husband trust you? (3); do you respect your husband? (2); important (2)
When you disagree with your husband, do you feel comfortable telling him that you disagree?	11 (23%)	Disagree with husband (3); comfort in disagreement (2); open communication (1); agree with husband (1); fear of beating (1)
Do you trust your mother‐in‐law to do things that are in your best interest?	28 (65%)^c^	Do you trust your mother‐in‐law for important matters? (16); do you rely on your mother‐in‐law in case of something important? (7); can you share with your mother‐in‐law? (3)
When you disagree with your mother‐in‐law, do you feel comfortable telling her that you disagree?	6 (14%)^c^	Disagree (2); comfort in disagreement (1)
Contraceptive method	30 (63%)	Not to conceive or to have a child (30)
Special foods for children (foods specifically designated for children and not consumed by adult household members)	18 (67%)^d^	Food items such as eggs and milk (12); liquid food (3)

*Note*. Respondents were asked to describe the question in their own words to measure understanding of key questions and terms.

a *n* = 47.

b *n* = 44.

c *n* = 43.

d *n* = 27.

For key terms used in the questions, the majority of participants understood terms as intended, with a few exceptions. The majority (60%) understood “contraceptive method” as “not having a child”; however, the original question had asked about specific methods. For the “special foods for children (i.e., foods specifically designated for children and not consumed by adult HH members)” question, two thirds of participants (66%) understood special foods as a variety of food items, including eggs, milk, fruits, and vegetables, despite special foods for children (e.g., infant cereals) being commonly available in Bangladesh. For questions regarding “milk/milk‐based products,” when primed to think about feeding their children over the age of 6 months, some participants interpreted it as “breast milk”; however, when primed to think about milk/milk‐based products to purchase, participants did not think about breast milk. Additionally, many participants interpreted the term “respect” in the intrahousehold relationships module as “honor.”

### 3.2 Comprehension: Recall period considered in responses

When asked what time period or event they recalled when responding to decision‐making questions, 85% referenced “a specific time when I was very ill” when asked about “when you are ill,” and 71% of participants responded, “child vaccination day” when asked about “whether your child gets vaccinations” (Table 3). For domains that referred to habitual decisions, more than half of the participants recalled a typical day or week when answering questions about food preparation (63%) and eating habits (52%), even though the fieldwork occurred shortly after Ramadan (fasting) and Eid (feasting) celebrations. Participants who did not reference a typical day or week reported that they referenced a specific event or day such as recalling to food preparation or eating habits during Ramadan or Eid.

**Table 3:** Recall and retrieval: Summary of responses to cognitive interview questions eliciting information on the time periods referenced during recall and perceived ability to recall experiences during key life cycle phases

*n* = 48		
Recall: Experiences or time periods referenced in responding to the question		
Decision‐making topic	Thought of a specific or habitual event *n* (%)	Example
Whether or not you consult a doctor or go to a clinic when you are ill?	41 (85%)	Specific time when you were very ill
How much you can rest when you are ill?	40 (83%)	Specific time when you were very ill
Whether or not to have a/another child?	40 (83%)	Before having first child; right now
Whether or not you use a contraceptive method?	36 (75%)	Talking to your husband about contraceptives
What foods to prepare every day?	30 (63%)	Typical day or week
What foods (available in the house) you can eat?	25 (52%)	Typical day or week
Whether your child is taken to a clinic or doctor is consulted when he/she is sick?	34 (71%)	A specific time when your child was sick
Whether your child gets vaccinations?	34 (71%)	Child vaccination day
How to feed your child when he/she is sick?	31 (65%)	A specific time when your child was sick
Retrieval: How well participants remembered decisions made during specific time periods		
	Remembered decisions well *n* (%)	
Decisions made during most recent pregnancy	38 (79%)	
Decisions made while breastfeeding youngest child	42 (88%)	

*Note*. For recall, participants were asked “Some people may think of specific experiences when they hear this question, or they may think about their life in general. Were you thinking of any of the following or something else?” For retrieval, participants were asked “Many people find it difficult to recall activities done a long time ago. How well do you remember the type of decisions that you made while [X]?”

### 3.3 Retrieval: Remembering decisions made during recent pregnancy and breastfeeding

When asked about decisions made during specific time periods (during their most recent pregnancy and breastfeeding their youngest child), the majority of participants stated that they remembered well the decisions made during their most recent pregnancy (79%) and while breastfeeding their youngest child (88%; Table 3). Participants mentioned that this time period was not too long ago for them to remember.

### 3.4 Judgment: Considering difficulty and comfort level of other community members

Almost all participants (94%) stated that they believed that other women in their community would not find the questions in these two pro‐WEAI modules difficult to answer. Reported reasons for finding the questions difficult included not being able to understand the question, needing time to think, being shy, and being uneducated. Participants stated, “As I felt [the question] was difficult, they will feel the same,” and “People who are not educated enough cannot understand properly” (Table 4, first set of columns).

**Table 4:** Judgment and response: Summary of responses to cognitive interview questions eliciting information on perceived level of comfort and difficulty experienced by self and potentially experienced by others

Decision‐making topic or question *n* = 48	Judgment		Difficulty		Discomfort	
	*n*	Example	*n*	Example	*n*	Example
Whether or not you consult a doctor or go to a clinic when you are ill	3 (6%)	Others may not understand/need time to think (1)	2 (4%)	I do not know what my mother in law thinks/minds if I tell something (1); cannot remember (1)	2 (4%)	Novelty of question/never thought in this way (2)
How much you can rest when you are ill?	2 (4%)	Others may not understand (1)	2 (4%)	Novelty of question/thinking (1); cannot measure amount of rest (1)	2 (4%)	I cannot explain my responses well (1)
Whether or not to have a/another child	—		4 (8%)	Husband is away, but it is usually a joint decision (1); conceiving is troublesome, so question was tough (1); I shall not take [another child], what will I tell? (1)	1 (2%)	Had trouble understanding the first time (1)
Whether or not you use a contraceptive method	—		2 (4%)	Never forget matter of the past. Never thought it seemed to be tough (1); I do not understand term “family planning” (1)	4 (8%)	Tough question/I did not understand (2); feeling ashamed (1)
What foods to prepare every day?	2 (4%)	Difficult (1); [others are] shy, uneducated (1)	1 (2%)	Difficult to think [of response] (1)	—	
Consulting a doctor/going to a clinic during you current or most recent pregnancy	—		—		1 (2%)	Household member (mother) present (1)
How much you worked during your current or most recent pregnancy?	1 (2%)	[Others are] uneducated (1)	—		—	
Whether your child visits the health clinic to see if he/she is growing well	1 (2%)	I had difficulty in understanding [question] (1); I cannot explain responses well (1)	—		—	
Who generally makes decisions about whether to purchase [product]?	—		1 (2%)	I cannot explain responses well (1)	1 (2%)	[Would] rather ask interviewer (1)
Do you trust your husband to do things that are in your best interest?	—		—		1 (2%)	I am afraid to tell (1)
When you disagree with your husband, do you feel comfortable telling him/her that you disagree?	2 (4%)	[The question is] difficult/I cannot understand (2)	5 (10%)	Tough [language] (4); understood [the question], but unable to tell because of fear (2)	3 (6%)	I am afraid of getting in trouble (2); it is a big question—I disliked the question (1)

*Note*. For judgment, participants were asked to determine if other women they knew would find it difficult to answer the questions and why. For responses, participants were asked if and why they found project‐level Women's Empowerment in Agriculture Index questions easy or difficult or felt uncomfortable.

### 3.5 Response: Difficulty and comfort level interpreting and answering questions

Overall, most participants (90%) did not cite difficulty or unease in answering any of the questions. Of those participants (10%) who did find questions difficult, participants stated that questions were hard to understand, they were unfamiliar with specific terms (e.g., contraceptive method), or they understood the question but had difficulty in formulating and explaining the responses well (e.g., “I cannot explain [my] responses well.”; Table 4, second set of columns).

Of the participants who found some questions as uneasy or uncomfortable (8%), they stated that they were not used to thinking in terms of the questions, grew tired of the question, or did not understand the question. For questions with domains of contraceptive method and comfort in telling husband if you disagree with him, participants felt they did not understand the question, felt ashamed to answer, or were in fear of getting in trouble if they answered. These was no pattern to how these responses were distributed across household type (Table 5, second set of columns).

**Table 5:** Context and experiences of making key decisions: Summary of responses to cognitive interview questions eliciting information on experiences having actually made these decisions

Decision‐making topic or question	Categorization of participants' responses to questions about decision‐making context^a^ and process^b^
Whether or not you consult a doctor or go to a clinic when you are ill	Feels self‐confident to take the decision; considers household income; consults with husband; considers her own health
Whether or not to have a/another child	Takes the decision with husband as it is both of their children; considers household income; considers the needs of her existing children
Whether or not to use a contraceptive method	Feels self‐confident to take decision; consults with husband
What foods to prepare every day?	Takes decision based on what her husband and children will eat; based on her own desire; is told by mother‐in‐law or husband what foods to prepare; consults with husband about food preparation for the day; prepares food items that her husband brings home
What foods available in the house you can eat?	Takes decision based on her desire; considers her own health
Consulting a doctor/going to a clinic during current or most recent pregnancy	Considers her own health and her child's health; consults with husband; asks for husband's permission regarding this decision; considers the advice of health worker; considers household income and consults with husband or mother‐in‐law
Consumption of eggs, milk and milk products, meat, poultry, and fish when child was being breastfed	Considers her own health and her child's health; consumes food items that her husband brings home; considers household income; considers the advice of her in‐laws and husband advises to eat certain foods.
Whether to take the child to a clinic or doctor when he/she is sick	Considers her child's health; considers household income and consults with husband about the situation; considers the advice of her husband, mother‐in‐law, and neighbours
Visiting health clinic to see if child is growing well	Feels self‐confident to take the decision as her child's well‐being is her responsibility; considers her child's health; considers the advice of her husband and mother‐in‐law; considers household income; considers the advice of doctor
Whether or not your child was offered milk/milk products to consume	Considers her child's health and food habits; feels self‐confident to take the decision as her child's well‐being is her responsibility; consumes food items that her husband brings home; consults her mother or family members
Whether or not your child was offered meat, poultry, or fish to consume	Considers her child's health and food habits; feels self‐confident to take the decision as her child's well‐being is her responsibility
Decision about breastfeeding	Considers her child's health as child's well‐being is her responsibility; feels self‐confident to take the decision about breastfeeding; consults other family members (e.g., sister‐in‐law)
Does your husband respect you?	Husband listens to her input; husband does not respect her
Do you trust your husband to do things that are in your best interest?	Trusts and relies on her husband; participant does not trust husband
When you disagree with your husband, do you feel comfortable telling him that you disagree?	Voices her disagreement to husband; participant does not disagree with husband
Does your mother‐in‐law respect you?	Mother‐in‐law loves and honours her; mother‐in‐law honours her less or does not honour her; participant and mother‐in‐law quarrel
Do you trust your mother‐in‐law to do things that are in your best interest?	Does not completely trust mother‐in‐law; relies on her mother‐in‐law
When you disagree with your mother‐in‐law, do you feel comfortable telling her that you disagree?	Voices her disagreement to mother‐in‐law; does not voice her disagreement with mother‐in‐law

*Note*. The content of this table is further elaborated in Table S2.

a Context question: “Please tell me what you were thinking while you answered this question.”

b Decision‐making process question: “I'd like you to tell me about a time when [e.g., you were ill]. If a decision was made to [e.g., consult a doctor/go to a clinic], how was the decision made [e.g., whether or not to consult a doctor or go to a clinic]?”

### 3.6 Context and decision‐making: Context of answers and decision‐making process regarding pro‐WEAI domains

When participants were asked to think back to their survey answers and elaborate on the experiences they referenced while responding to survey questions, approximately half of the participants provided meaningful responses that revealed additional context surrounding decision‐making. Common themes included the following: She expressed confidence that her understanding of the situation is better than others; she believed another individual, primarily her husband, or mother‐in‐law, knew better about the situation than she herself did; she contributed to decisions related to household finances; she believed that her decision or opinion would be viewed as acceptable and supported by the household; and she was considering her own health or her child's health and future well‐being. The context that participants referenced differed based on the decision being made. For example, for decisions regarding one's own need for rest while sick, participants often explained that they understood the situation better than anyone else. However, for decisions regarding visiting the doctor, some participants cited deferring to their husband's decision because he had control of the household finances (Table 5).

Some participants in households with a coresident mother‐in‐law mentioned consulting her or other in‐laws about decisions, such as when the child became sick or who to the leave the child with when she went out, more than participants in the other household types. These participants often stated, “As the sister‐in‐law is responsible for our household, so she will take decision” and “Father‐in‐law knows well about doctors.”

Participants in households with a migrant husband often described making decisions alone in the absence of her husband or consulting others before making decisions. For example, one participant from a migrant‐sending household mentioned she made decisions alone more often when her husband was away because he was not there to make the decision. Others in this household type mentioned consulting their mothers‐in‐law or other family members in the household before making decisions in the absence of their husband, stating, “Then I take decision by discussing with my mother‐in‐law.” Some participants mentioned that they would call their husband on the phone to discuss bigger decisions, such as sending the child to school, but typically, the participant would be left to make the final decision. In this context, sole decision‐making may not necessarily entail agency but rather a lack of support or that they relied on nonhousehold members to help make decisions.

## 4 DISCUSSION

The results of the cognitive interviewing for the pro‐WEAI health and nutrition and intrahousehold modules revealed that questions were generally well understood by participants and the majority of participants interpreted the questions and key terms as intended. Participants stated that they thought of specific events for most questions and thought of habitual events for questions related to food preparation and eating habits. Additionally, participants stated that they could remember the decisions made during specific time frames, such as their most recent pregnancy and while breastfeeding their child. Though not an objective evaluation of recall during these periods, it suggests that participants are able to understand and formulate responses to these questions based on their perceived experiences.

The results also revealed insights into how women formulated their responses and the context of the experiences they referenced when answering the survey questions, which enriched the interpretation of the survey responses. For example, among the women who stated that she and her husband make decisions together, some of the women mentioned that their lack of control of household finances required them to consult their husbands before making a decision to go to the doctor. Other participants who had a migrant husband mentioned making decisions alone, when no one else was around to make decisions, and consulting family members, friends, and neighbours, to help make decisions. This finding suggests that although participants with migrant husbands may report making decisions alone, it may be out of necessity, and so the woman seeks the input of her support system for decision‐making. Researchers who design and interpret survey questions and responses should consider that the responses, when interpreted at face value, may reveal only part of the larger context of decision‐making.

Despite that the questions generally were well understood, the results also revealed potential shortcomings, which could lead to survey responses that do not accurately reflect participants' experiences. When interpreting questions and formulating the responses, participants who misinterpreted questions often focused on the second part of the question (a specific decision that might be made). Some key terms were also not interpreted as intended, such as “contraceptive methods,” “special foods for children,” “milk/milk products,” and “respect.” The majority of women stated that they did not have difficulty or feel uncomfortable in interpreting and answering questions, nor did they think that other women in the community would find the questions difficult or uncomfortable. Of the women who found questions difficult or uncomfortable, topics such as “contraceptive method” and “comfort in telling husband if you disagree with him” were the most problematic. Social context may be a factor that drives timid responses, and women who are shy or less educated may have difficulty responding to questions as an experience of disempowerment. Thus, response bias may not just arise due to cognitive processes but could indicate that women who are less empowered may have diminished ability to respond to questions about empowerment and decision‐making.

### 4.1 Recommendations for module revision

To rectify these shortcomings, we propose specific recommendations that could improve the pro‐WEAI health and nutrition and intrahousehold relationships modules. It is recommended to reorder the clauses in some questions to ask the part eliciting a response after the decision‐making topic is introduced. For example, “Who in the household generally makes decisions about [ACTIVITY]?” should be rewritten as, “When decisions are made about [ACTIVITY], who normally takes the decision?” Additionally, “Who would you prefer make the decisions about [ACTIVITY]?” should be rewritten as, “When decisions are made regarding [ACTIVITY], who would you prefer make the decision?” In this way, participants can focus on the question of interest, instead of focusing on the decision itself.

For complex questions that require interpretation at multiple levels and may be sensitive for some participants, we recommend asking the question as two different questions. For example, “When you disagree with your husband/mother‐in‐law, do you feel comfortable telling him/her that you disagree?” should be rewritten in two parts as: “Do you ever disagree with your husband/mother‐in‐law?” [If yes] “Do you feel comfortable telling him/her that you disagree?” Administering the question separately may improve participants' interpretation of the question and ease their discomfort, resulting in stronger responses. Additionally, questions that focus on sensitive topics, such as contraceptive use, should be included later in the survey. This will allow the interviewer to build rapport with the participant and for the participant to grow accustomed to the types of questions. When asking about potentially sensitive topics, interviewers should be trained to observe non‐verbal cues that potentially indicate that the respondent is shy or feeling uncomfortable about the topic. By noticing these cues, interviewers can encourage honest responses from more disempowered women, which may help reduce response bias.

For terms that were misinterpreted, adding specific examples may facilitate understanding. For example, “contraceptive method” can be elaborated with specific examples; “special foods for children” can include item names; and “milk/milk products” can be clarified as “other than breast milk.” For the term, “respect,” some participants interpreted the term as “honor” in Bengali. Although both “respect” and “honor” may be interpreted similarly and used interchangeably in Bengali and English, we recommend providing specific guidance on translation in the instruments documentation. To address the fact that some respondents may be seeking input from household members who reside elsewhere (e.g., migrant husbands), we encourage users of the modules to include context‐specific response categories for nonhousehold members (e.g., spouse via telephone).

### 4.2 Strengths and limitations

This study was conducted in a context where the pro‐WEAI is being used to evaluate the impact of nutrition‐sensitive agriculture programmes. This allowed us to work with a data firm that was familiar with the module. Additionally, the findings from this study, which will inform revisions of the pro‐WEAI health and nutrition and intrahousehold modules, are directly applicable to the impact evaluations of these programmes, as well as other measures of women's empowerment that are in use or development. The specific findings may not be fully generalizable outside of the study sites, as additional shortcomings could be identified in other contexts. However, many of the issues raised may be relevant elsewhere. Finally, the length of the cognitive interview may have increased participant burden. This was remedied by dividing questionnaire administration over 2 days. We recommend that others who use this methodology consider dividing the module and assigning different sections to different participants.

## 5 CONCLUSION

Although the accuracy of measuring women's empowerment has improved, survey instruments have been used routinely without a full assessment of their measurement properties. This practice can lead to inaccurate or biased responses and misleading conclusions (Miedema et al., 2018; Yount, 2005; Yount et al., 2016). Given the complexity of women's empowerment as a multidimensional construct, applying rigorous approaches to the development of such instruments is critical. Cognitive interviewing is one such approach that should be a routine part of questionnaire design and testing. Results of this study revealed shortcomings in the pro‐WEAI health and nutrition and intrahousehold modules and revealed specific ways to improve the instrument. Refining these instruments will allow researchers to collect better quality data on women's empowerment, enhancing our capacity to monitor progress on the Sustainable Development Goals.

## CONFLICTS OF INTEREST

The authors declare that they have no conflicts of interest.

## CONTRIBUTIONS

JH and AH conceptualized the study and designed the questionnaires. AH led the fieldwork, conducted the analysis, and wrote the first draft of the manuscript. LJH provided guidance for the analysis and support for the writing of the manuscript. JH contributed to the revisions of the writing of the manuscript and provided overall guidance. KY provided technical guidance and support for the writing of the manuscript. All authors have read and approved the final manuscript.

## Supporting information

Table S1. Survey items in the pro‐WEAI nutrition and health module and intrahousehold module

Table S2. Context and experiences of making key decisions: Responses to cognitive interview questions eliciting information on experiences having actually made these decisions (extension of information provided in Table 5)

Table S3. Codebook
