# Supplementary material for: Cognitive interviewing to improve women's empowerment questions in surveys: Application to the health and nutrition and intrahousehold relationships modules for the project‐level Women's Empowerment in Agriculture Index
Source: Matern Child Nutr. 2019 Aug 14;16(1):e12871. doi: 10.1111/mcn.12871 (PMC7038906; doi:10.1111/mcn.12871)
Supplement: Supplementary file 1 — Table S1. Survey items in the pro‐WEAI nutrition and health module and intrahousehold module [file MCN-16-e12871-s001.docx]

**Online Supplementary Table 1.** Survey items in the pro-WEAI nutrition and health module and intrahousehold module

| **HEALTH AND NUTRITION MODULE: WOMEN'S HEALTH AND NUTRITION** | | |
| --- | --- | --- |
| **Questions** | **Activities** | **Response options** |
| **Q1.** Who in the household generally makes decisions about [ACTIVITY]?  **Q2.** To what extent do you feel you can participate in decisions regarding [ACTIVITY] if you wanted to?  **Q3.** Who would you prefer make the decisions about [ACTIVITY]? | A. Whether or not you consult a doctor or go to a clinic when you are ill B. How much you can rest when you are ill C. Whether or not you have a/another child D. Whether or not you use a contraceptive method E. What foods to prepare every day F. What foods (available in the house) you can eat G. Whether you consulted a doctor or went to a clinic during your current or most recent pregnancy* H. How much you worked during your current or most recent pregnancy* I. How much you could rest during your current or most recent pregnancy* J. Whether you could eat eggs during your current or most recent pregnancy* K. Whether you could consume milk or milk products during your current or most recent pregnancy* L. Whether you could eat meat, poultry, or fish during your current or most recent pregnancy* M. How much you worked when your youngest child was being breastfed* N. How much you could rest when your youngest child was being breastfed* O. Whether you could eat eggs when your youngest child was being breastfed* P. Whether you could consume milk or milk products when your youngest child was being breastfed* Q. Whether you could eat meat, poultry, or fish when your youngest child was being breastfed* | **Q1.** Enter up to three member IDs  (linked to names in household roster via a computer-assisted personal interviewing)  **Q2.** (Select one)  Not at all  To a small extent  To a medium extent  To a high extent  **Q3.** Enter up to three member IDs  (linked to names in household roster via a computer-assisted personal interviewing) |
| **HEALTH AND NUTRITION MODULE: CHILD HEALTH AND NUTRITION** | | |
| **Questions** | **Activities** | **Response options** |
| **Q1.** Who in the household generally makes decisions about [ACTIVITY]?  **Q2.** To what extent do you feel you can participate in decisions regarding [ACTIVITY] if you wanted to?  **Q3.** Who would you prefer make the decisions about [ACTIVITY]? | A. Whether your child is taken to a clinic or a doctor is consulted when he/she is sick B. Whether your child gets vaccinations C. Whether your child visits the health clinic to see if he/she is growing well D. How to feed your child when he/she is sick E. Who will care for your child when you need to go outside the home for an extended period of time F. Sending your child to school G. Whether your child is offered eggs to eat** H. Whether your child is offered milk or milk products** I. Whether your child is offered meat, poultry, or fish** J. Whether to breastfeed your child*** K. When to stop breastfeeding your child*** L. When to start introducing foods and liquids (other than breastmilk) to your child M. Whether your child is fed special foods for children that programs or health workers tell you should be consumed | **Q1.** Enter up to three member IDs (linked to names in household roster via a computer-assisted personal interviewing)  **Q2.** (Select one)  Not at all  To a small extent  To a medium extent  To a high extent  **Q3.** Enter up to three member IDs (linked to names in household roster via a computer-assisted personal interviewing) |
| **HEALTH AND NUTRITION MODULE: HEALTH PRODUCTS** | | |
| **Questions** | **Products** | **Response options** |
| **Q1**. Who in the household generally makes decisions about whether to purchase [PRODUCT]?  **Q2.** If you need [PRODUCT], are you usually able to acquire it by some means (e.g., purchasing or cultivating it yourself or having someone do it for you)? | A. Small quantities of food, for example smaller than 5 kg B. Large quantities of food, for example larger than 5 kg C. Eggs D. Milk or milk products E. Meat, poultry, or fish (including organ meats) F. Special foods for children than programs or health workers tell you should be consumed G. Any nutritious foods that a program or health worker told you to consume H. Medication, vitamins, or supplements for children I. Medication, vitamins, or supplements for yourself J. Clothing for children K. Clothing for yourself L. Toiletries, such as soap and toothpaste | **Q1.** Enter up to three member IDs  (linked to names in household roster via a computer-assisted personal interviewing)  **Q2.** (Select one)  Yes  No  Not applicable |
| **INTRAHOUSEHOLD RELATIONSHIPS MODULE** | | |
| **Questions** | **Relation** | **Response options** |
| **Q1.** Do you [NAME] respect your [RELATION]?  **Q2.** Does your [RELATION] respect you?  **Q3.** Do you trust your [RELATION] to do things that are in your best interest?  **Q4.** When you disagree with your [RELATION], do you feel comfortable telling him/her that you disagree? | A. Husband  B. Mother-in-law**** | **For all Questions** (Select one)  Most of the time  Sometimes  Rarely  Never |
| *Only asked to women who have been pregnant or given birth in the last 2 years | |  |
| **Only asked to women who have a child aged 6 months or older | |  |
| ***Only asked to women who have a child aged 2 years or younger | |  |
| ****Only asked to women with a mother-in-law residing in the same household | |  |
