# Supplementary material for: Cognitive interviewing to improve women's empowerment questions in surveys: Application to the health and nutrition and intrahousehold relationships modules for the project‐level Women's Empowerment in Agriculture Index
Source: Matern Child Nutr. 2019 Aug 14;16(1):e12871. doi: 10.1111/mcn.12871 (PMC7038906; doi:10.1111/mcn.12871)
Supplement: Supplementary file 2 — Table S2. Context and experiences of making key decisions: Responses to cognitive interview questions eliciting information on experiences having actually made these decisions (extension of information provided in Table 5) [file MCN-16-e12871-s002.docx]

**Online Supplementary Table 2:** Context and experiences of making key decisions: Responses to cognitive interview questions eliciting information on experiences having actually made these decisions (extension of information provided in Table 5)

| **Decision-making topic or question** | **Categorization of participants’ responses to questions about decision-making context**^a^ **and process**^b^ | **Example Responses** |
| --- | --- | --- |
| Whether or not you consult a doctor or go to a clinic when you are ill? | Feels self-confident to take the decision; Considers household income; Consults with husband; Considers her own health. | “I am educated, I understand well” |
| How much you could rest during illness | Considers her own health | “I was physically weak and tired, then I took rest.” |
| Whether or not to have a/another child | Takes the decision with husband as it is both of their children; Considers household income; Considers the needs of her existing children. | “It would be better if two of us take decision.” |
| Whether or not to use a contraceptive method? | Feels self-confident to take decision; Consults with husband | “I told it by thinking that I discussed about this matter with my husband.” |
| What foods to prepare every day | Takes decision based on what her husband and children will eat; based on her own desire; Is told by mother-in-law or husband what foods to prepare; Consults with husband about food preparation for the day; Prepares food items that her husband brings home | “I choose/decide food items to cook that are good for my health and health of my husband, my child, and my mother-in-law.” |
| What foods available in the house you can eat | Takes decision based on her desire; Considers her own health | “I can eat when I wish to eat, decision is taken in this way.” |
| Consulting a doctor/going to a clinic during current or most recent pregnancy | Considers her own health and her child’s health; Consults with husband; Asks for husband’s permission regarding this decision; Considers the advice of health worker; Considers household income and consults with husband or mother-in-law. | “I know good and bad of my physical condition. It was my decision, have told by thinking that.”  “I will not be able to go unless my husband permit, have answered by thinking this matter.” |
| Amount worked during your current or most recent pregnancy | Considers her own health and her child’s health during decision-making | “Both my child and I will be well.” |
| Amount of rest during current or most recent pregnancy | Considers her own health and her child’s health during decision-making | “[I] have thought, if I take rest my body will be kept well, [my] child will be healthy. [The] body does not stay well all the time.” |
| Consumption of eggs, milk and milk products, meat, poultry, and fish during your current or most recent pregnancy | Considers her own health and her child’s health during decision-making | “[My] child will be healthy if [I eat] milk and egg.” |
| Amount worked when child was being breastfed | Considers her own health and her child’s health during decision-making | “I do work as I wish, I do look after my child in my way which I have told by thinking.” |
| Amount of rest when child was being breastfed | Considers her own health and her child’s health during decision-making | “Generally, I take decision by myself regarding of taking rest- when I will take rest and when I will not take rest.” |
| Consumption of eggs, milk and milk products, meat, poultry, and fish when child was being breastfed | Considers her own health and her child’s health; Consumes food items that her husband brings home; Considers household income; Considers the advice of her in-laws and husband advise to eat certain foods. | “Mother [I] understands well regarding which food would not be harmful to child’s health, have answered by thinking of this matter.” |
| Whether to take the child to a clinic or doctor when he/she is sick | Considers her child’s health; Considers household income and consults with husband about the situation; Considers the advice of her husband, mother-in-law, and neighbors. | “When my child was seriously ill, then I myself have taken decision that I would take my child to Doctor.” |
| Whether to get child vaccinated | Considers her child’s health during decision-making; Considers the advice of her husband and mother-in-law during decision-making; Considers the advice of her neighbors, peers, doctors, and health workers during decision-making | “I have taken decision to give vaccination so that my child healthy.” |
| Visiting health clinic to see if child is growing well | Feels self-confident to take the decision as her child’s well-being is her responsibility; Considers her child’s health during decision-making; Considers the advice of her husband and mother-in-law during decision-making; Considers household income during decision-making; Considers the advice of doctor during decision-making | “It is my responsibility whether my child is growing up or not. So I take decision in this matter.”; “She is our child. It is good if decision is taken by both of us together in close cooperation.” |
| How to feed your child during when he/she was sick | Feels self-confident to take the decision as her child’s well-being is her responsibility; Consults the doctor during decision-making | “I understand well by feeding what /which food my child would be felt good, have answered by thinking this matter.” |
| Who would care for your child when you needed to go outside the home for extended period of time | Feels self-confident to take the decision and consults with her own mother, mother-in-law, or neighbor; Consults her husband and mother-in-law during decision-making | “I will tell her grandmother, I have work in the outside. Take care of my child, I am going.” |
| Sending child to school | Considers her child’s future during decision-making; Feels self-confident to take the decision about her child’s education; Consults with husband during decision-making | “She/he [child] will become a good human being if she/he is sent to school”. |
| Whether or not your child was offered milk/milk products to consume | Considers her child’s health and food habits; Feels self-confident to take the decision as her child’s well-being is her responsibility; Consumes food items that her husband brings home; Consults her mother or family members. | “Child will [not] get nutrition unless [he/she is fed] milk, egg, fish, meat etc. I myself give these food to my child.” |
| Whether or not your child was offered meat, poultry, or fish to consume | Considers her child’s health and food habits; Feels self-confident to take the decision as her child’s well-being is her responsibility | “I think regarding feeding of my child. Because I am mother. I understand good and bad of child. For this reason, I told.” |
| Decision about breastfeeding | Considers her child’s health as child’s well-being is her responsibility; Feels self-confident to take the decision about breastfeeding; Consults other family members (e.g., sister-in-law). | “I have told because it was my decision to breast-feed my child.” |
| Decision to stop breastfeeding | Common practice is that breastfeeding will end when child is age 2 to 2.5, so there is no need to make the decision. | “Breast milk will have to stop after 2-2.5 years, So the decision is mine.” |
| Introducing foods and liquids | Considers her child’s health during decision-making; Feels self-confident to take the decision | “I will understand when it could be good to feed extra food to child. Because the child is mine.” |
| Feeding child special foods for children that programs or health workers discuss that should be consumed | Considers her child’s health during decision-making; Considers the advice of health worker during decision-making; Feels self-confident to take the decision and child’s well-being is her responsibility; Considers household income during decision-making | “Child’s brain will develop if it can be given to the child. I have taken decision thinking of this matters.”; “Thinking of my husband. Because it is he who earns money.” |
| Do you respect your husband? | Participant honors and listens to her husband | “I always obey my husband’s words/instruction.” |
| Does your husband respect you? | Husband listens to her input; Husband does not respect her. | “My husband gives attention to what I tell.”; “I have none [respect].” |
| Do you trust your husband to do things that are in your best interest? | Trusts and relies on her husband; Participant does not trust husband | “If I tell him to bring important things, then he brings for me.”; “If I tell anything, he does not behave well, scolds me, so I do not trust him.” |
| When you disagree with your husband, do you feel comfortable telling him that you disagree? | Voices her disagreement to husband; Participant does not disagree with husband | “I can convince my husband. He pays heed to my word.”; “I never disagree.” |
| Do you respect your mother in law? | Participant honors and listens to her mother in law | “I honor my mother-in-law. When she tells anything, then I pay heed to her word.” |
| Does your mother in law respect you? | Mother-in-law loves and honors her; Mother-in-law honors her less or does not honor her; Participant and mother in law quarrel | “She respects me.”; “She honors me less.”; “Often she quarrels with me.” |
| Do you trust your mother in law to do things that are in your best interest? | Does not completely trust mother-in-law; Relies on her mother-in-law | “[I] do not rely on [her] completely.”; “I do not tell all matters, [as] I am daughter of other [household].”; “I shared many things to her as I believe in her.” |
| When you disagree with your mother in law, do you feel comfortable telling her that you disagree? | Voices her disagreement to mother-in-law; Does not voice her disagreement with mother-in-law | “If I make my mother in law understand, then she understands and pays heed to my word.”; “I do not tell. If I tell, there will be quarreling.” |

^a^ Context question: “Please tell me what you were thinking while you answered this question”

^b^ Decision-making process question: “I’d like you to tell me about a time when [e.g., you were ill]. If a decision was made to [e.g., consult a doctor/go to a clinic], how was the decision made [e.g., whether or not to consult a doctor or go to a clinic]
