# Supplementary material for: Cognitive interviewing to improve women's empowerment questions in surveys: Application to the health and nutrition and intrahousehold relationships modules for the project‐level Women's Empowerment in Agriculture Index
Source: Matern Child Nutr. 2019 Aug 14;16(1):e12871. doi: 10.1111/mcn.12871 (PMC7038906; doi:10.1111/mcn.12871)
Supplement: Supplementary file 3 — Table S3. Codebook [file MCN-16-e12871-s003.docx]

| Code | Description | Example |
| --- | --- | --- |
| Together | A woman states that a decision is taken jointly by the woman and her husband | “The decision that I will take, it to be taken [by] discussion with my husband, then it would be good.” |
| Cannot take decision alone | A woman states that a decision cannot be taken by herself and she must consult with her husband or another before a decision is made | “I could not be able to take decision alone without my husband’s opinion.” |
| Health | A woman’s consideration for her own health or her child’s health when making a decision | “These have to be eaten to keep the body healthy. The health will remain good.” |
| Finances | A woman takes into account her control of household finances when making a decision | “How will I go to doctor if my husband does not give money?” |
| Self-confidence | A woman’s confidence that her understanding of the situation is better than that of others | “I am educated, I understand well.” |
| Others know better | A woman’s belief that another individual, particularly her husband or mother-in-law, knows about the situation better than the woman does; Someone other than the woman makes the decision  Other individuals include: husband, in-laws (mother-in-law, father-in-law, sister-in-law), mother, neighbors/peers, doctor/healthcare worker | “Husband would understand what is good or bad for his wife.” |
| Decision-acceptance | A woman’s belief that her decision and opinion would be viewed as acceptable | “We both have to decide. The decision of [my own] will not do.” |

**Online Supplementary Table 3. Codebook**
